# An Older Adult With Abdominal Fullness

**DOI:** 10.1016/j.acepjo.2026.100454

**Published:** 2026-06-30

**Authors:** Cheng-Yu Lai, Yu-Chin An

**Affiliations:** Department of Emergency Medicine, Tri-Service General Hospital, National Defense Medical University, Taipei, Taiwan

A 71-year-old man with prior left hemicolectomy presented with abdominal fullness after swallowing a sour plum 2 days earlier, accompanied by mild nausea and poor oral intake. Examination revealed mild periumbilical tenderness without peritoneal signs. Laboratory testing showed leukocytosis (11.2 × 10^3^/μL) with neutrophil predominance (89.4%). Abdominal radiography was nondiagnostic. Bedside point-of-care ultrasound demonstrated dilated, fluid-filled proximal small bowel loops with prominent plicae circulares (keyboard sign) (Fig 1) and an arch-like intraluminal hyperechoic lesion with posterior acoustic shadowing, raising concern for mechanical small bowel obstruction (Fig 2).

**Figure 1:**
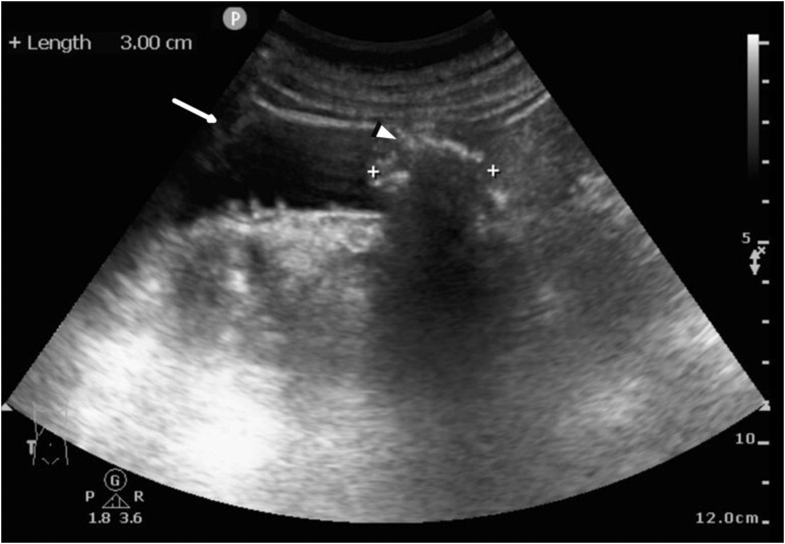
Point-of-care ultrasound demonstrating dilated, fluid-filled small bowel loops (arrow) with intraluminal arch-like hyperechoic lesion (arrowhead).

**Figure 2:**
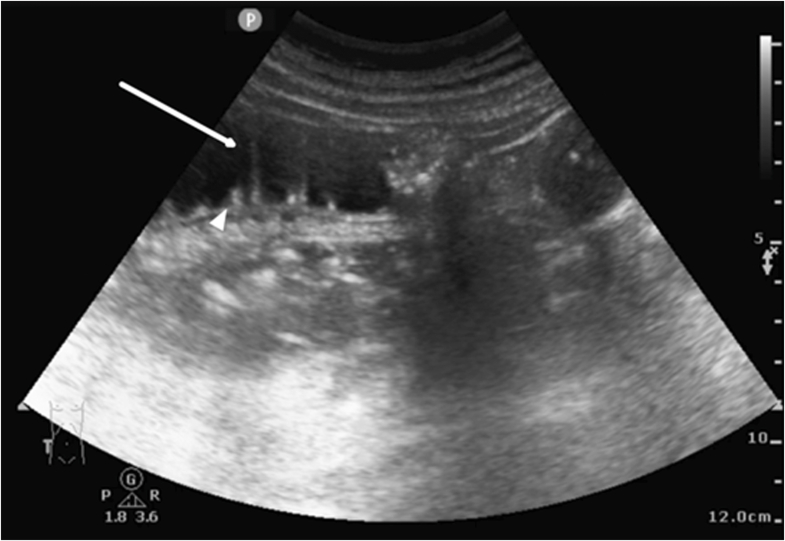
Point-of-care ultrasound demonstrating prominent plicae circulares (arrow) within a fluid-filled small bowel loop, known as keyboard sign (arrowhead).

## 1 Diagnosis

### 1.1 Phytobezoar-induced small bowel obstruction

**Figure 3:**
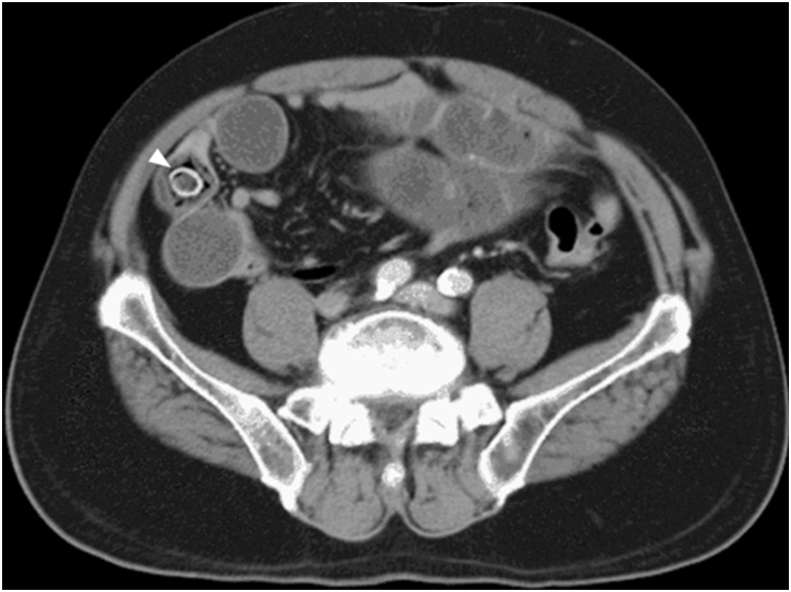
Abdomen computed tomography axial view demonstrating small bowel obstruction with a transition point in the right lower quadrant, associated with mottled intraluminal gas and a rim-calcified intraluminal lesion (arrowhead) proximal to the transition zone.

**Figure 4:**
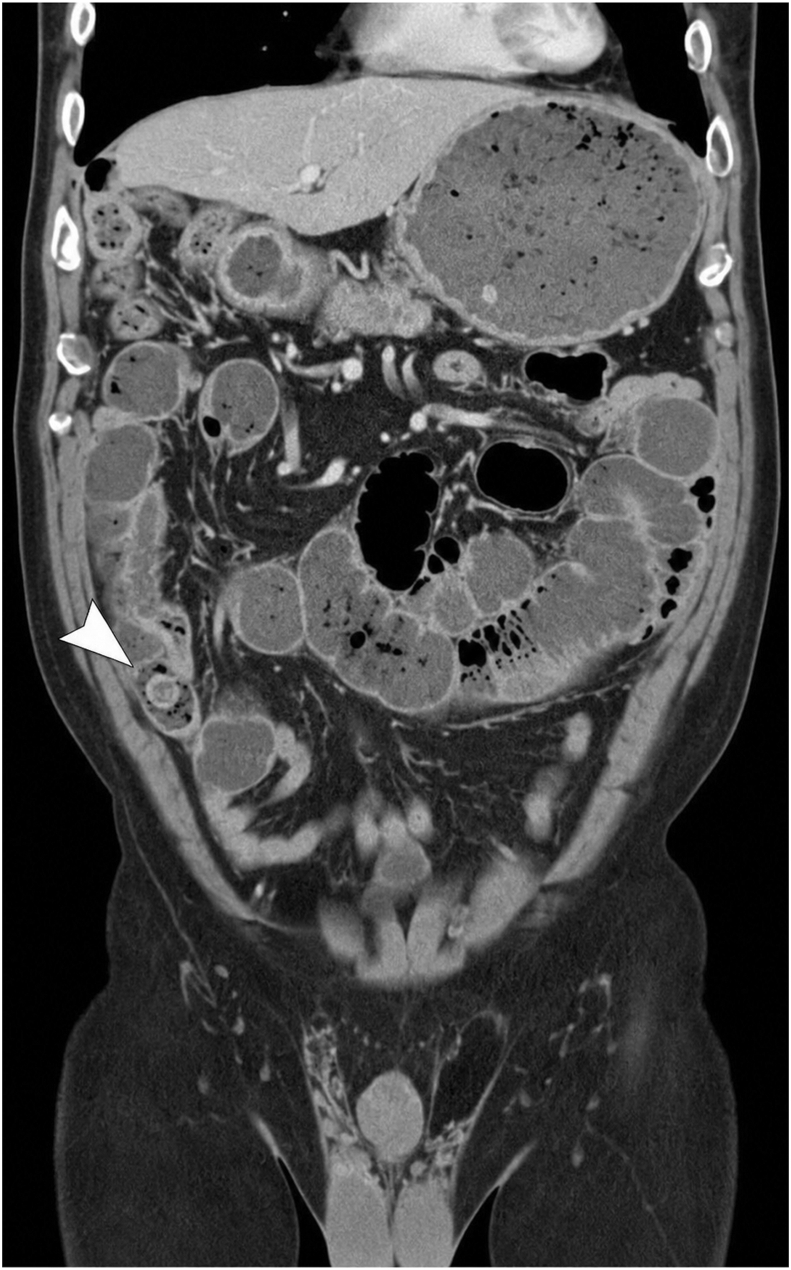
Abdominal computed tomography coronal view showing dilated proximal small bowel loops and a transition point in the right lower abdomen caused by a rim-calcified intraluminal lesion (arrowhead).

Small bowel obstruction with imaging findings consistent with phytobezoar. Contrast-enhanced abdominal computed tomography demonstrated a right lower quadrant transition point with mottled intraluminal gas and a rim-calcified intraluminal lesion proximal to the transition zone, supporting phytobezoar-related obstruction.^1^^,^^2^ (Figs 3 and 4) Bezoars are an uncommon but recognized cause of mechanical small bowel obstruction and may show characteristic intraluminal echogenicity on ultrasound and mottled gas-containing intraluminal masses on computed tomography.^1^^,^^2^ Point-of-care ultrasound can facilitate early bedside recognition of small bowel obstruction when plain radiography is inconclusive.^3^ The patient improved with bowel rest, nasogastric decompression, and supportive care, and follow-up water-soluble contrast study confirmed resolution without surgery.

## Funding and Support

By *JACEP Open* policy, all authors are required to disclose any and all commercial, financial, and other relationships in any way related to the subject of this article as per ICMJE conflict of interest guidelines (see www.icmje.org). The authors have stated that no such relationships exist.

## Conflict of Interest

All authors have affirmed they have no conflicts of interest to declare.
